# 450-nm blue diode laser: a novel medical apparatus for upper tract urothelial lesions

**DOI:** 10.1007/s00345-023-04647-x

**Published:** 2023-10-13

**Authors:** Dali Jiang, Guoxiong Liu, Bing Yang, Haoming Niu, Hengtong Fan, Zejun Ren, Liyue Mu, Xiaofeng Xu, Ximin Qiao, Kaijie Wu, Dalin He

**Affiliations:** 1 Department of Urology, First Affiliated Hospital of Xi’an Jiaotong University, #277 Yanta West Road, Xi’an, 710061 People’s Republic of China; 2 Department of Urology, Xianyang Central Hospital, Xianyang, 712000 People’s Republic of China; 3 Xi’an Blueray Technology Co., Ltd., Xi’an, 710061 People’s Republic of China

**Keywords:** 450-nm blue laser, UTUCs, Endoscopic surgery, Preclinical research

## Abstract

**Objective:**

To explore the feasibility, safety and effectiveness of the 450-nm blue diode laser (BL), novel blue laser in the treatment of upper tract urothelial carcinomas (UTUCs) and other lesions in a porcine model.

**Material and methods:**

For in vitro experiment, the ureter tissue was vaporised and coagulated with BL, green-light laser (GL) and Ho:YAG laser (Ho). The efficiency, width and depth of vaporisation, and depth of coagulation were recorded and compared. For in vivo experiments, four swines weighing 70 kg were used. In the acute group, different modes of operations were performed to evaluate the thermal damage, perforation and bleeding. In the chronic group, the overall appearance of the ureter and laser wound healing were observed by the naked eyes and H&E staining 3 weeks after surgery.

**Results:**

In in vitro study, the BL showed a higher efficiency of tissue vaporisation and less tissue coagulation for fresh ureter compared to GL and Ho. In the in vivo study, the power of BL set at 7 W was better, and the thickness of thermal damage varied with different surgery types in the range of 74–306 μm. After 3 weeks, the wound healed well static in vaporisation (SV), moving vaporisation (MV) and H&E staining indicated mucosal healing rather than scar healing.

**Conclusion:**

5–10W blue diode laser achieved a higher efficiency of tissue vaporisation and less tissue coagulation in a porcine model, indicating its potential application in the endoscopic surgery of UTUC as an optional device with high performance and safety.

## Introduction

Upper tract urothelial carcinomas (UTUCs) account for 5–10% of all urothelial carcinomas [1], and nephroureterectomy with bladder-cuff resection is considered the gold standard for the management of UTUC. However, for some patients with a solitary kidney or poor surgical tolerance, the selection of endoscopic tumour resection/ablation is also a good choice to maximise kidney function and prolong life [2–4].

For instruments of endoscopic treatment, there are rigid, semirigid and flexible endoscopies with different techniques, such as fulguration, laser vaporisation and cryoablation. Taking advantage of the accurate resection and excellent haemostasis of lasers, various lasers with different laser fibres have been applied to treat UTUC, such as the 1064-nm neodymium laser (Nd:YAG), 1.9-μm thulium–YAG laser (Tm:YAG), 2.1-μm holmium–YAG laser (Ho:YAG), and 532-nm lithium triborate laser (green-light laser, GL) [5–7].

The efficacy of the laser-tissue interaction is determined by the wavelength of the laser and the diameter of the fibre core. Given the complications of the endoscopic management of UTUC [4], evaluating and mastering the effectiveness and safety of different lasers to the renal pelvis and ureter appear to be particularly important. In our previous studies, we demonstrated that the 450-nm blue diode laser with high vaporisation efficiency and minimal thermal damage could be used in the transurethral resection of non-muscle invasive urothelial bladder cancers [8]. However, its application in the endoscopic treatment of UTUC remains unknown. This study aimed to explore its feasibility, safety and effectiveness in treating upper tract urothelial lesions in a porcine model compared to two other commonly used lasers (i.e. GL and Ho).

## Materials and methods

### Laser system and experimental platform

This study was approved by the Ethics Committee of Xi’an Jiaotong University and the Hospital’s Institutional Animal Care and Use Committee. The 450-nm blue diode laser (Blueray Medical, Xi’an, China) was used as described in our previous study [8]. The green-light laser system and Ho:YAG laser system were from Realton (Beijing, China) and Lumenis (California, United States), respectively. The experimental platform was operated on a three-axes custom-made linear stage with X–Y motorised moving and Z manually moving. The two computer-controlled electrical step-motors independently controlled speed and position of X and Y axes. On Z axis stage, the fibre holder mounted was used to manually adjust the distance from the fibre tip to the tissues [8, 9].

### Model of porcine ureteral tissues for ex vivo study

Fresh kidneys and ureteral tissues were obtained from a local slaughterhouse. The ureteral tissue was cut longitudinally into a section 5 cm long and 2.5 cm wide, according to our previous study [5]. To evaluate the laser–ureter tissue interaction, the power of the laser setting was tested in the ex vivo experiment as follows: 5 W, 10 W, 15 W and 20 W; the working distances from the fibre tip to ureteral tissues were set as 0.5 mm, 1 mm, 2 mm and 3 mm; and the moving speeds of the fibre tip were set as 0.5 mm/s, 1 mm/s and 2 mm/s. Every set was repeated at least three times. A Vernier calliper was used to measure the depth and width of the laser vaporisation, and photographs of a gross specimen were taken by Canon G12. The formula of the vaporisation efficiency was length (10 mm) × width (mm) × depth (mm)/(10 mm/2 mm/s). The vaporised tissues were fixed in 4% paraformaldehyde solution for 48 h, embedded in paraffin, sectioned into slices, stained with haematoxylin and eosin, and imaged with an Olympus SZ61 to measure the thickness of coagulation and carbonization.

### Study design of the in vivo procedure

Two male and two female swines weighing 70 kg were used. One male and one female swines were randomly used in the acute group and the chronic group, respectively. Induction of anaesthesia for the experimental swine was performed with serazine hydrochloride injection and 3% pentobarbital sodium by intramuscular injection. The maintenance of anaesthesia was utilised with enflurane and disoprofol. The swine was supine, and a flexible ureteroscope (Olympus Urf-p6) was used through a trocar at the anterior wall of the bladder with a 5 mm incision. The flexible ureteroscope was used in the bladder, ureter and pelvis, and then a laser fibre was placed in the target tissues with a laser power setting of 7 W. The experiment was performed in the different parts of each ureter, and repeated on the left and right sides of the ureter in each swine. After the operation, the specimens were obtained for H&E staining.

In the acute group, the perforation, intraoperative bleeding and thickness of the coagulation were recorded during the whole procedure. According to clinical experience and the literature of laser treatment of UTUC [10, 11], for different size, shape and location of the ureteral tumour, we designed three experimental methods of the static vaporisation (SV), moving vaporisation (MV), and cyclic vaporisation (CV). Three types of BL surgery, including static vaporisation (SV), moving vaporisation (MV), and cyclic vaporisation (CV), were performed in three different sites of each ureter, and then repeated on the left and right sides of the ureter in each swine. So each experiment of SV, MV and CV in different ureters was repeated four times. After 3 h, the swines were euthanized, and the specimens were taken for photographs and fixed with 4% paraformaldehyde for H&E staining.

In the chronic group, the types of surgery were SV, MV and CV, each experiment in different ureters was repeated four times. A double J tube (6Fr size) was inserted into the ureteral lumen. After the laser surgery, the swines were fed for 3 weeks. During the first week, oral antibiotics of levofloxacin capsules were used to prevent infection. Three weeks later, urologic ultrasound and ureteroscopy were used to examine wound healing and hydronephrosis. The intact ureter was exposed, and the renal ureter appearance was observed and photographed. Keeping away from the healing site, the ureter and pelvis were dissected longitudinally, and wound healing was observed by the naked eyes and then photographed. The ureter tissue was fixed in 4% paraformaldehyde, and H&E staining was carried out.

### Statistical analysis

The continuous variables were reported as the mean ± standard deviation (SD) using SPSS 18.0 software (IBM Corporation, Armonk, NY). The statistical chart was generated using GraphPad Prism 5.

## Results

### Ex vivo analysis of the laser–ureteral tissue interaction with BL, GL and Ho

To evaluate which laser was the best choice for the treatment of upper tract urothelial lesions and select the appropriate laser power, speed and working distance for the in vivo study, various parameters of 450-nm blue diode laser, 532-nm green-light laser and 2.1-μm Ho:YAG laser were tested in the ex vivo experiments.

**Fig. 1:**
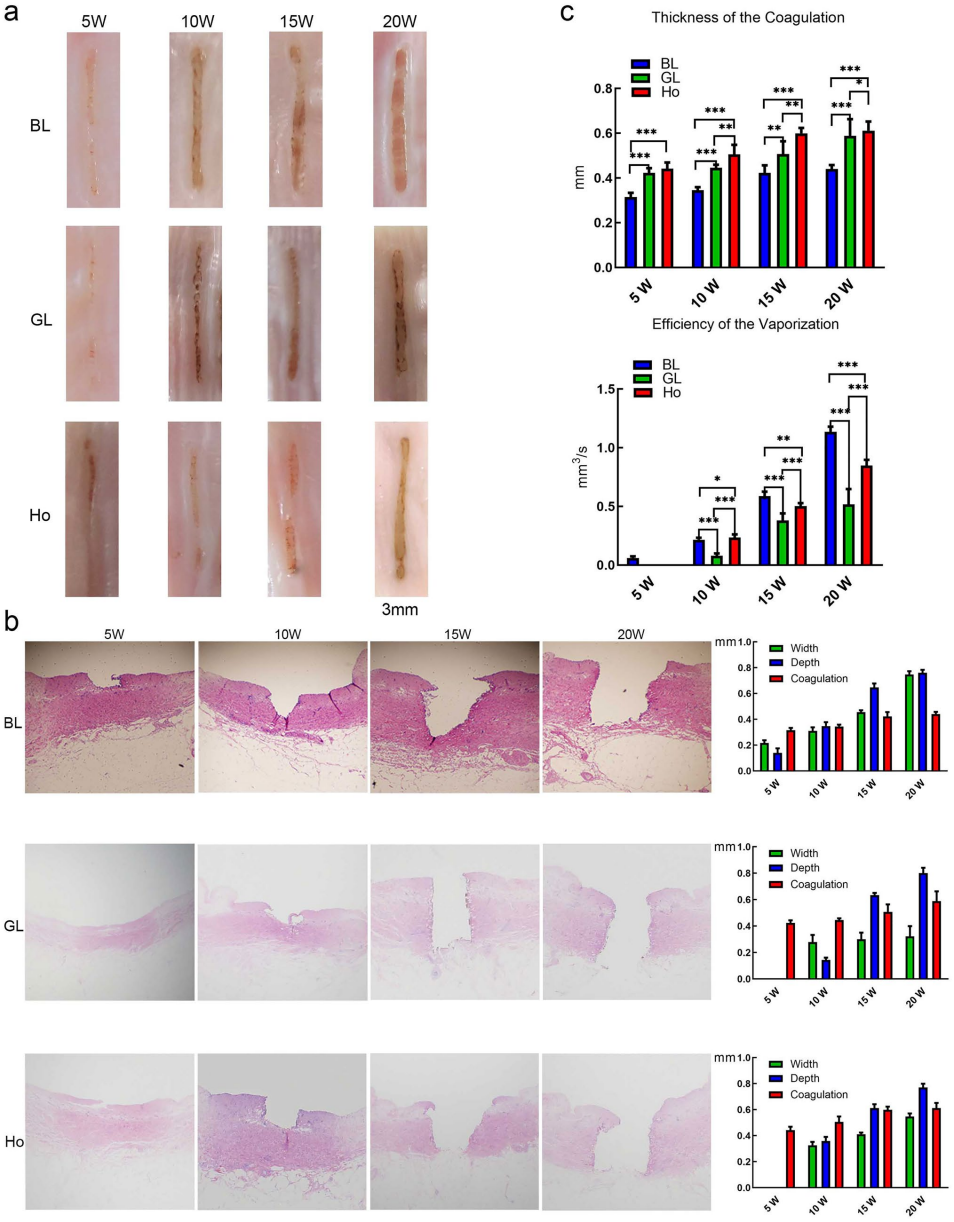
A ex vivo vaporisation of ureteral tissues by BL, GL and Ho at a power of 5–20 W, **a** Speed of 2 mm/s and a working distance of 2 mm. Gross view of ureteral tissues after vaporisation of the three lasers at different powers. **b** The histological results by H&E staining of ex vivo vaporisation of ureteral tissues by BL, GL and Ho at a power of 5–20 W, at a speed of 2 mm/s and a working distance of 2 mm. **c** Statistical analysis of laser vaporisation efficiency. **a–b** Thickness and efficiency of BL, GL and Ho. *p < 0.05, **p < 0.01, ***p < 0.001

As shown in Fig. 1, we observed that the depth and width of laser vaporisation, but not the thickness of coagulation, increased as the power increased. However, the efficacy of laser vaporisation decreased as the laser working speed increased. The width of vaporisation increased as the working distance, but the depth decreased as the working distance increased. For coagulation, if the vaporisation was sufficient, the thickness of coagulation had no significant change.

Furthermore, we found that the blue laser vaporisation was efficient for fresh ureters and the coagulation was stable when the laser working speed and distance were 2 mm/s and 2 mm. As shown in Fig. 1a, b, the widths for the powers of 5 W and 10 W were 0.22 ± 0.02 mm and 0.31 ± 0.03 mm, respectively, and the depths were 0.14 ± 0.04 mm and 0.35 ± 0.03 mm, respectively, and the coagulations were 0.32 ± 0.02 mm and 0.35 ± 0.01 mm, respectively. As the power increased, the laser penetrated the entire ureter, and the width, depth and coagulation were 0.46 ± 0.02 mm, 0.75 ± 0.02 mm and 0.65 ± 0.03 mm for 15 W and 0.76 ± 0.02 mm, 0.42 ± 0.03 mm and 0.44 ± 0.02 mm for 20 W, respectively.

For tissue coagulation of different powers from BL, GL and Ho, as shown in Fig. 1 c, the coagulation from BL was the lowest. From 5 to 20 W, the coagulation of BL, GL and Ho ranged from 0.31 to 0.44 mm, 0.42 to 0.58 mm and 0.44 to 0.61 mm, respectively. For tissue vaporisation efficiency, as shown in Fig. 1c, BL was the highest. From 5 to 20 W, the efficiency of BL, GL and Ho ranged from 0.06 to 1.14 mm^3^/s, 0.08 (10 W) to 0.52 mm^3^/s and 0.23 (10 W) to 0.85 mm^3^/s, respectively.

### In vivo procedures of the laser–ureteral tissue interaction with BL

According to the ex vivo experimental results of BL, GL and Ho, BL seemed to be the most advantageous laser surgical system for UTUC. The 7 W of BL with a better vaporisation effect and safety was selected for the in vivo procedure. Then we used BL to complete the in vivo experiment. Accordingly, the power of the BL was set at 7 W, and the operating distance from the laser fibre tip to the target tissue in the endoscopy was 1–2 mm.

**Fig. 2:**
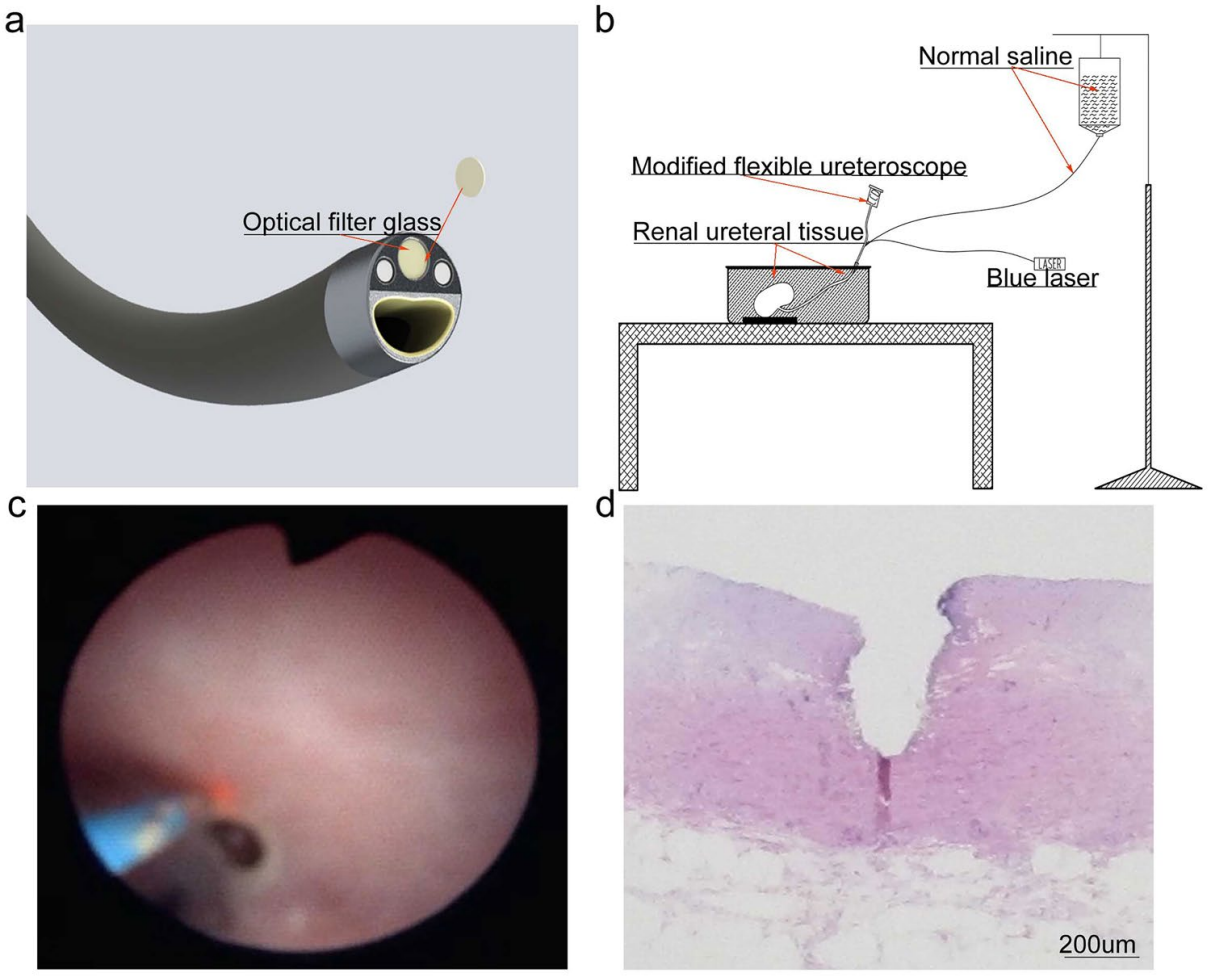
Modifying electronic flexible ureteroscopy lens and simulated operation in vitro. **a** Electronic flexible ureteroscopy lens and filter; **b** Schematic diagram of simulated ureteral vaporisation by blue laser assisted by the modified soft lens; **c** Screenshot of simulated operation; **d** H&E staining of vaporised ureteral tissue

As shown in Fig. 2a–c, a clear surgical field was obtained from the modified electronic flexible ureteroscope during ureter vaporisation in three different types of endoscopic surgery by the blue laser. H&E staining indicated that blue laser vaporisation had reliable cutting security with limited damage to the ureteral mucous layer only (Fig. 2d).

**Fig. 3:**
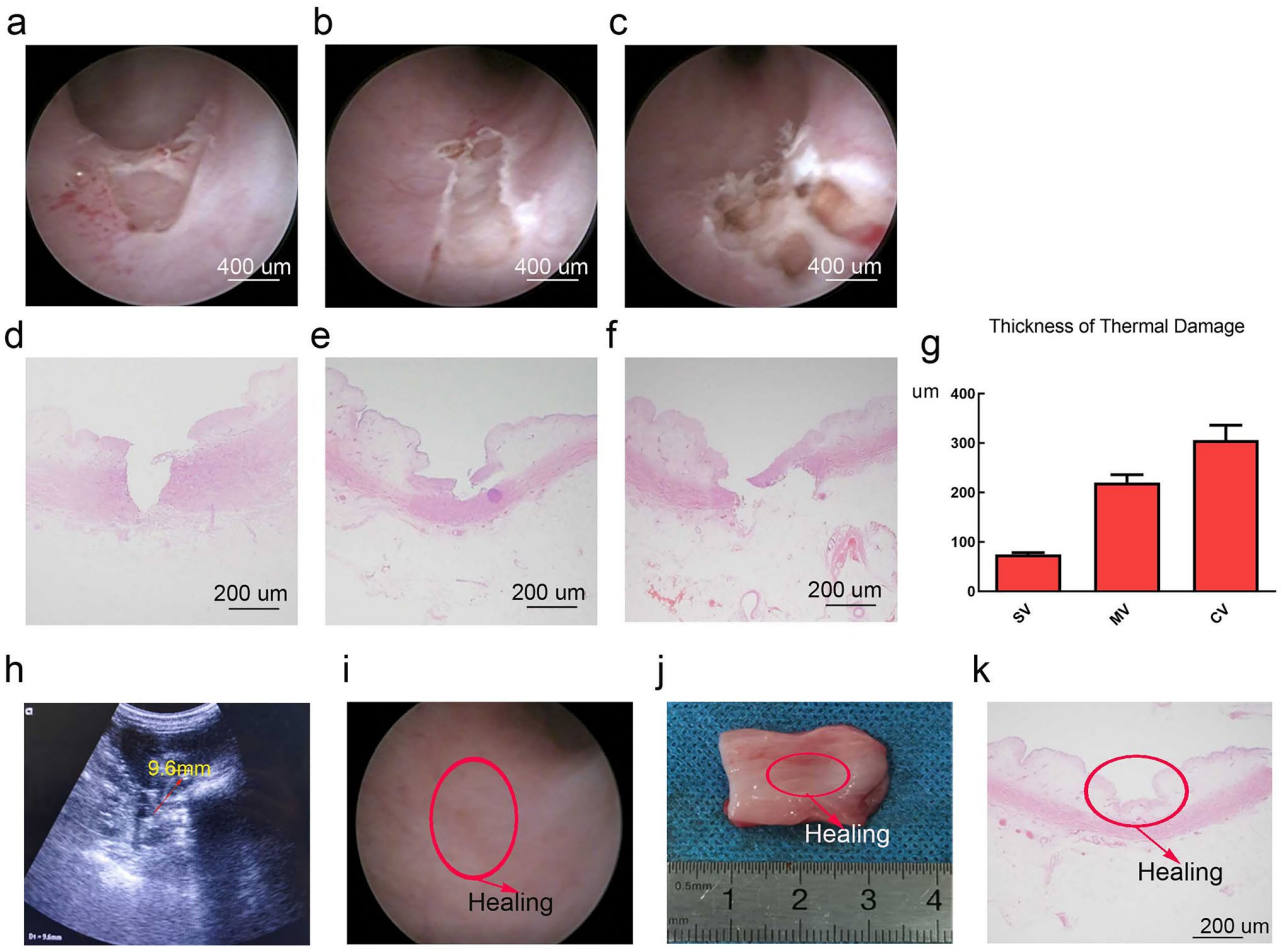
The acute effects of a blue diode laser on ureteral tissue vaporisation in the upper urinary tract with three different surgical strategies in vivo and the chronic effects of a blue diode laser on ureteral tissue vaporisation in the upper urinary tract with three different surgical strategies after 3 weeks. **a** Static vaporisation (SV); **b** Moving vaporisation (MV); **c** Cyclic vaporisation (CV). **d**–**f** The corresponding histological images by H&E staining of ureteropelvic tissues by SV, MV and CV. **g** Statistical analysis of the thickness of thermal damage. **h** Little hydronephrosis by urological ultrasound. **i** Ureter healing by ureteroscopy. **j**–**k** Gross view and histological images by H&E staining of ureteropelvic tissue healing after laser vaporisation

In the acute group, no perforation or bleeding were observed. As shown in Fig. 3a–g, the thickness of thermal damage in SV, MV and CV was 0.074 ± 0.004 mm, 0.22 ± 0.016 mm and 0.306 ± 0.03 mm, respectively. Therefore, we should pay attention to the performance of CV using the blue laser to treat upper tract urothelial lesions, which was much more dangerous. In the chronic group, as shown in Fig. 3h–k, little hydronephrosis was observed by urological ultrasound after 3 weeks of laser surgery, whilst ureteroscopy showed that the ureter healed well without obvious scarring after laser vaporisation.

## Discussion

According to previous studies, different kinds of lasers have been utilised in the treatment of UTUC, such as the 532-nm KTP laser, thulium laser (Tm:YAG) and holmium laser (Ho:YAG) [6, 12–14]. The purpose of various laser studies and applications was to explore its feasibility, safety and effectiveness. In this study, we demonstrated a new laser for the potential use in the treatment of UTUCs. Our data revealed that the BL showed a higher efficiency of tissue vaporisation and less tissue coagulation for ureter compared to GL and Ho. Taken together, these findings suggest that the blue diode laser achieved potential application in the endoscopic surgery of UTUC as an optional device with high performance and safety.

The wavelength of the laser determines the efficacy of the laser–tissue interaction, so different kinds of lasers produce different effects. The blue diode laser has several potential advantages in the treatment of different diseases due to its higher absorption by haemoglobin, its more efficient vaporisation and resulting cutting effect, and the comparatively moderate thickness of a thermal damage layer [8, 9, 15–17]. Herein, we have first reported a novel 450-nm blue diode laser to treat upper tract urothelial lesions ex vivo and in vivo in a porcine model.

Emiliani et al. reported that vascular structures are observed at a depth of 0.4 mm, the adventitia starts 0.6–0.7 mm from the lumen, including macrovasculature around the peri-ureter and the muscular layer starts approximately 0.1–0.2 mm from the lumen [18]. In our ex vivo experiments when the laser power was 5 W and 10 W, its vaporisation depth was 0.14 ± 0.04 mm and 0.35 ± 0.03 mm, respectively, and the maximal coagulation was 0.32 ± 0.02 mm and 0.35 ± 0.01 mm. In the in vivo experiments, the thermal layer was much less, which was 0.074 ± 0.004 mm, 0.22 ± 0.016 mm and 0.306 ± 0.03 mm, respectively in the SV, MV and CV. Although muscularis mucosae layer vaporisation was observed in the SV, MV and CV, there was no exudation or penetration and wound healing was not affected during the chronic study. Therefore, these data strongly suggest that the blue laser is safe for ureteropelvic surgery. From the efficacy and coagulation of different powers and different laser types, the power of the 5–10 W blue laser seems to be suitable for the clinical treatment of UTUC patients in the future, which balances the efficacy and safety of the blue laser vaporisation to treat urothelial lesions.

An in vitro study showed that the temperature of ureteral calculi treated with a holmium laser reached 70 ℃ in the absence of flushing water and less than 39 ℃ in the presence of flushing water [19]. Regarding whether the blue laser can cause high-temperature damage to the ureter, such as the holmium laser in the ureter, our previous study showed that the water temperature only rose to 42 ℃ when the blue laser operated continuously for 30 min without flushing [20]. Studies have shown that cells can only be damaged if they are exposed to ambient temperatures of 43 ℃ for a long time [21]. This result is related to the fact that water does not absorb the blue laser energy. Therefore, it is safe and reliable to use the blue laser for the surgical treatment of intraureteral lesions.

The coagulation thickness of the blue laser vaporisation is thinner than that of GL and Ho, indicating that it has more accurate vaporisation effect. The ureteral tissue is thin. If the laser with thick coagulation layer is used to vaporise the tumour, it is easy to perforate the tissue to leak urine. To achieve favourable oncological outcomes, as reported by Takashi Yoshida et al. [22], under photodynamic diagnosis (PDD) guidance, the treatment of UTUCs by the blue laser might also have a well tumour control with the ideal scope of tissue coagulation.

However, this study also had some limitations. For example, the changes in ureteral lumen temperature were not monitored during the blue laser vaporisation in vivo. Also, the animal number is too small, and we will increase the number of animals and compare the blue laser to GL and Ho lasers in vivo in our further experiments. More evidence is required to support the potential application of blue lasers in the endoscopic treatment of UTUC.

## Conclusion

Our study is the first to recommend the novel 450-nm blue diode laser surgery system as a safe and efficient medical equipment compared to other lasers in the treatment of UTUC. We conclude that the low power of the blue laser with proper working distance and speed can achieve accurate cutting and satisfactory ureteropelvic healing. However, training is required for healthcare providers who will use the laser, and further animal and clinical studies are warranted for continued verification of this therapeutic approach.

## Data Availability

The data that support the findings of this study are available from the corresponding author upon reasonable request.
